# Development of a Web-Based Peer Support Program for Family Caregivers of Ventilator-Assisted Individuals Living in the Community: Protocol for a Pilot Randomized Controlled Trial

**DOI:** 10.2196/11827

**Published:** 2019-02-06

**Authors:** Marina B Wasilewski, Mika Nonoyama, Craig Dale, Douglas A McKim, Jeremy Road, David Leasa, Roger Goldstein, Louise Rose

**Affiliations:** 1 Lawrence S Bloomberg Faculty of Nursing University of Toronto Toronto, ON Canada; 2 Faculty of Health Sciences University of the Ontario Institute of Technology Oshawa, ON Canada; 3 Rehabilitation and Sleep Laboratory The Ottawa Hospital Ottawa, ON Canada; 4 The Lung Centre Vancouver General Hospital Vancouver, BC Canada; 5 London Health Sciences Centre London, ON Canada; 6 West Park Healthcare Centre Toronto, ON Canada; 7 Florence Nightingale Faculty of Nursing, Midwifery & Palliative Care King’s College London London United Kingdom; 8 Sunnybrook Research Institute Sunnybrook Health Sciences Centre Toronto, ON Canada; 9 Prolonged Ventilation Weaning Centre Michael Garron Hospital Toronto, ON Canada

**Keywords:** intervention, caregivers, peer support, mechanical ventilation

## Abstract

**Background:**

Across Europe, Canada, Australia, and the United States, the prevalence of home mechanical ventilation (HMV) prevalence is 6.6-12.9 per 100,000. At-home ventilator-assisted individuals (VAIs) are often vulnerable and highly comorbid, requiring complex care. In Canada, most VAI care is provided by family, leading to poor health-related quality of life and increased caregiver burden. No supportive interventions or peer support programs are tailored to VAI caregivers. Owing to the financial, geographic, and time limitations, Web-based support delivery may especially meet VAI family caregiver needs. We have developed a peer mentor training and Web-based peer support program for VAI caregivers including information-sharing, peer-to-peer communication, and peer mentorship.

**Objective:**

Study Stage 1 aims to (1) evaluate the face and content validity of the peer mentor training program and (2) investigate participant satisfaction. Study Stage 2 aims to evaluate (1) the feasibility of participant recruitment and Web-based program delivery; (2) acceptability, usability, and satisfactoriness; (3) experiences of caregivers and peer mentors with the Web-based peer support program; and (4) effect of the Web-based peer support program on caregiver health outcomes.

**Methods:**

Study Stage 1: We will train 7 caregivers to act as peer mentors for the Web-based peer support program trial; they will complete questionnaires rating the utility of individual training sessions and the training program overall. Study Stage 2: We will recruit 30 caregiver peers for a pilot randomized controlled trial of the 12-week Web-based peer support program using a waitlist control; the program includes private chat, a public discussion forum, and weekly moderated chats. Caregiver peers will be randomized to the intervention or waitlist control group using a 1:1 ratio using Randomize.net. Both groups will complete pre- and postintervention health outcome questionnaires (ie, caregiving impact, mastery, coping, personal gain, positive affect, and depression). Caregiver peers in the intervention arm will only complete a program evaluation and will be invited to participate in an interview to provide insight into their experience. Peer mentors will be invited to participate in a Web-based focus group to provide insight into their experience as mentors. We will judge the feasibility per the number of recruitment and program delivery goals met, use analysis of covariance to compare health outcomes between intervention and control groups, and analyze qualitative data thematically.

**Results:**

Peer mentor training was completed with 5 caregivers in July 2018. To date, 2 caregivers have beta-tested the website, and the Web-based peer support program trial will commence in September 2018. Results are expected by early 2019.

**Conclusions:**

This study will result in the production and initial evaluation of a rigorously developed, evidence- and stakeholder-informed Web-based peer training and peer support program for caregivers of VAIs residing at home.

**International Registered Report Identifier (IRRID):**

PRR1-10.2196/11827

## Introduction

### Family Caregivers of Ventilator-Assisted Individuals

Prevalence rates for ventilator-assisted individuals (VAIs) living at home are increasing globally. The 2005 Eurovent Study estimated the prevalence of home mechanical ventilation (HMV) at 6.6 per 100,000 population across 16 European countries [1]. Comparative research from Australia and New Zealand indicated a minimum prevalence of HMV of 9.9 and 12.0 per 100,000, respectively [2]. Recent data from Canada and the United States indicate that 12.9 and 6.6 per 100,000 population, respectively, receive HMV [3,4]. Given the unique and complex care needs of VAIs [3], family caregivers play an essential role in sustaining the stable environment that enables them to live at home [5,6]. If family caregivers are unable to support VAIs to remain in the home, the only alternative is institutionalization [7]. Several studies have highlighted that caregiving can increase the burden and decrease the caregiver health-related quality of life [8]. A study focusing on caregivers of VAIs with progressive neuromuscular disease, a common indication from HMV, described a negative impact on both physical and emotional caregiver health, with the initial transfer home perceived as extremely stressful [5]. These findings emphasize the growing acceptance that family caregiving is a serious public health issue requiring intervention [9].

### Support From Other Caregivers

Peer support comprises (1) emotional support (expressions of caring, empathy. and reassurance); (2) informational support (advice, suggestions, factual input, and feedback); and (3) affirmational support (affirmation of feelings and behaviors, reassurance that frustrations can be managed) [10]. Peers—individuals who have experienced the same health problem and have similar characteristics as the support recipients—can be a key source of support for family caregivers [11]. Peer support improves the health-related quality of life and well-being by decreasing feelings of isolation, improving mood, buffering stress, creating a sense of empowerment, and increasing self-efficacy in various patient and caregiver populations [12-14]. The lack of peer support has been shown to predict distress [15]. Several peer support models exist including in-person, peer-to-peer matching, and online support [16].

### Online Peer Support Provision

Within the broader family caregiver population, geographic limitations, time constraints and a lack of respite have been cited as reasons that caregivers do not utilize in-person support groups [17]. In addition, the economic burden of diseases that leads to mechanical ventilation frequently make attendance at in-person meetings cost-prohibitive [18]. Consequently, many caregivers are turning to the internet as an avenue for social support [19]. Over the past decade, the number of Web-based health interventions providing a broad array of family caregiver supports in a cost-effective, accessible, and convenient fashion have increased exponentially [20]. Within these Web-based programs, peer-to-peer communication is a particularly critical element of support, with qualitative findings indicating that caregivers highly appreciate and benefit from interaction with similar others [20]. Therefore, peer support delivered using Web-based modalities is especially well-tailored to fit the demanding nature of the VAI caregiver role.

### Development of a Web-based Peer Support Program for Caregivers of Ventilator-Assisted Individuals

Rising numbers of VAIs living at home [3], the burden of family caregiving [5,9], the proven benefit of peer support in various caregiving populations, and the lack of existing social support for caregivers of community-based VAIs [6] suggest an urgent need for peer support programs for these family caregivers. Although peer support interventions have been shown to be effective in a number of illness populations, there are currently no studies of family caregivers of VAIs in the home. As peer support can take many forms [16] and multicomponent Web interventions (ie, including several support features— informational links, chat, and discussion forum) tend to be more effective in reducing caregiver burden [21,22], we will develop, validate, and conduct feasibility evaluation of an Web-based peer support program entailing elements of informational support, peer-to-peer interaction, as well as peer-mentoring.

### Overall Study Objective

This study aims to develop a comprehensive Web-based peer support program for VAI caregivers, including peer-mentoring, and explore the feasibility of the program.

## Methods

### Study Stage 1: Peer Mentor Training

#### Objectives

##### Primary Objective

The primary objective is to evaluate the utility of the peer mentor training program.

##### Secondary Objective

The secondary objective is to investigate participant satisfaction with the peer mentor training program.

#### Procedure

We have adapted a peer *training* program developed by St. Jude’s Research Hospital (Memphis, TN, USA) for parents caring for a child with cancer [23], also recommended as a valuable resource by an existing peer support tool kit used for parents of technology-dependent children [16]. We selected this training program because of its focus on mentorship skill development (rather than peer-matching process and logistics) and the publicly available recorded training sessions. To ensure that our adapted *training* program has the face and content validity for our home VAI caregiver population, we will ask a minimum of 2 home VAI caregivers and 1 clinician that manages this patient population to review and make suggestions regarding content, structure, and delivery.

The original St. Jude’s peer *training* program consists of 4 in-person sessions and our Web-based mentor training program will reflect this. The four 1-hour sessions will cover the following topics: (1) peer mentorship basics (eg, family-centered care, where or how to obtain educational materials specific to HMV); (2) mentoring skills (eg, active listening and sharing stories); (3) boundary-setting (eg, mentor’s boundaries and value of boundaries); and (4) mentorship at the end-of-life, emergency situations, and wrap-up (eg, unique end-of-life circumstances, red flags, when to call for help, debriefing about training, and next steps for participating in evaluation). During each of the sessions, the trainer will go over the material and incorporate short “break-out sessions” that will allow participants to pause, reflect, and discuss new concepts and skills.

We will host mentor training sessions using GoToMeeting to facilitate Web-based attendance. GoToMeeting allows for high-definition video and high-quality audio, compatibility with desktops or tablets or phones, screen sharing, and video recording. This latter feature will enable recording of the training sessions and archiving on a secure server to inform future iterations of the training program.

#### Participants

The inclusion criteria for peer mentors are as follows: (1) age ≥18 years; (2) previous or current family caregiver for a community-residing VAI; (3) able to speak and read English; (4) access to a computer (with video and microphone) and a high-speed internet connection; and (5) available for training sessions. The exclusion criteria are as follows: (1) currently experiencing severe depression as indicated by a score of ≥10 on the Centre for Epidemiological Studies Short Depression scale during recruitment screening [24].

#### Recruitment

We will recruit caregivers from the provincial Ventilation Equipment Pool (Kingston, ON, Canada), and the long-term and home ventilation clinics of West Park Healthcare Centre (Toronto, ON, Canada). We will seek snowball referrals from clinician experts, professional societies, patient advocacy groups (eg, Muscular Dystrophy Canada), and through Twitter.

We will recruit 5-10 peer mentors for training. Purposive sampling based on the following criteria will be used to ensure sample diversity: (1) ventilator type (invasive: n=2; noninvasive: n=5); (2) diagnostic category [rapidly progressing disease (amyotrophic lateral sclerosis): n=2; nonrapidly progressing disease (Guillain-Barré syndrome, Myasthenia Gravis, and postpolio syndrome): n=2; variably progressing disease (muscular dystrophy): n=2]; (3) relationship to care-recipient (spouse: n=2; child: n=2; parent: n=1); and (4) sex (male: n=2; female: n=5).

#### Data Collection

Prior to training, we will ask peer mentors to complete a demographic questionnaire and rate their general health status (on a scale of 1-5; 1=very good, 5=Poor). Before and after the training program participation, we will ask peer mentors to complete the Mentoring Skills Inventory [25]; this questionnaire asks peer mentors to indicate whether they are very comfortable (“V”), moderately comfortable (“M”), or uncomfortable (“U”) with 12 mentoring skills (eg, brokering relationships; coaching; goal-setting; managing conflict; providing and receiving feedback) [25,26]. After each training session, we will ask peer mentors to complete a short questionnaire rating the extent to which they agree with a series of questions about the design, content, instruction, and utility of the training session on a 5-point Likert scale (1=strongly disagree; 5=strongly agree). In addition, they will be asked to comment on what they benefited from most, what was unclear, and what would benefit from additional content. Any mentor(s) identified as needing clarification or additional training will be followed up with on an individual basis. We will ask peer mentors to complete an end-of-training questionnaire rating their satisfaction with the training overall (eg, objectives clearly defined and trainers knew material) on a 5-point Likert scale (1=strongly agree; 5=strongly disagree). Furthermore, they will be asked to comment on which training sessions they found most or least informative and to provide any suggestions for future iterations of the peer mentor training program.

#### Data Analysis

We will report descriptive statistics from individual training session questionnaires and the end-of-training questionnaires, including counts and proportions for categorical data and means and SDs (or medians and interquartile ranges), depending on the distribution of continuous data.

### Study Stage 2: Web-based Peer Support Program

#### Objectives

##### Primary Objective

The primary objective is to evaluate the feasibility of trial recruitment and program delivery according to *a-priori* definitions described below and including user ratings of acceptability, usability, and satisfaction.

##### Secondary (Exploratory) Objectives

The secondary objectives are to explore (1) caregiver health outcomes (ie, caregiving impact, mastery, coping, personal gain, positive affect, and depression) before and after participation in the Web-based peer support program and (2) the experiences with the program from the perspective of caregiver peers and peer mentors.

#### Trial Design

We will conduct a pilot randomized controlled trial (RCT) of the 12-week Web-based peer support program with waitlist control. Research Ethics Board approval was received from the University of Toronto in May 2017, where the research is being conducted.

#### Study Setting

While the online peer support intervention is Web based, the study is being hosted at the University of Toronto. Recruitment is limited to Canadian caregivers of VAIs.

#### Eligibility Criteria

The inclusion and exclusion criteria for the peer mentor training also apply to participants for the Web-based peer support program feasibility RCT except needing to be *currently* providing care to a VAI living at home.

### The Web-based Peer Support Program (Intervention)

#### Technical Development

The end goal for this development project was to create a “social-network” style website (akin to Facebook). This website would also need to digitally capture and record all interactions between participants. To provide all the functionality needed for this study (including interaction data capture), we found that nonproprietary “off-the-shelf” software programs or templates were insufficient. Therefore, we used a hybrid of “existing-base-software” coupled with code or programming designed and integrated into this base software, making the functionality of this website unique.

#### Content and Design

The content and design of the Web-based peer support program were informed by a scoping review led by the first author [20], a local peer support tool kit [16], and a provincial peer support program [27]. The 12-week Web-based peer support program entails the following: (1) informational resources (links to relevant websites and resources—eg, national disease and caregiving organizations); (2) discussion forum open to caregiver peers and peer mentors enabling asynchronous contact; (3) weekly chat (live 1-hour forum for discussing a specific topics—eg, self-care, illness management moderated by the research team); and (4) private messaging, including audio, video, or text chat, allowing participants one-on-one or select group interaction with other caregiver peers or peer mentors. Private messaging is hidden from other participants but accessible to the research team for monitoring purposes. Every participant (peer mentors and caregiver peers) will create a personal profile with information about their caregiving situation (eg, who are or were caring for, ie, spouse, child, duration of care, diagnosis, etc). Based on the peer mentor profiles, caregiver peers will have the opportunity to access mentors they believe are well suited to address their support needs, questions, and concerns.

### Primary Outcome

Our primary outcome is the feasibility of trial recruitment and program delivery. The feasibility will be assessed on the basis of compliance with the protocol represented by the following criteria:

1. The proportion of peer mentors participating weekly in any program element (ie, discussion forum, private chat, or live chat) for, at least, 8 out of the 12 program weeks: ≥60% of peer mentors
2. The proportion of caregiver peers participating weekly in any program element (ie, discussion forum, private chat, or live chat) for, at least, 8 out of the 12 program weeks: ≥60% of peer mentors
3. Discussion forum usage: ≥50% participants (peer mentors and caregiver peers) posting each week
4. Live weekly chat usage: ≥50% participants (peer mentors and caregiver peers) joining each week
5. The frequency of weekly mentor contacts: ≥25% of mentors receive, at least, 1 message each week
6. The proportion of peer mentors contacted: ≥50% of mentors contacted, at least, once during the 12-week program
7. The proportion of caregiver peer participants who contacted a mentor: ≥50% of caregiver peer participants during the 12-week program
8. Attrition rates: ≤30% caregiver peer participants withdrawing from the study before completion of postintervention questionnaires

We have selected the following decisions to determine the feasibility [28,29]:

- 0-2/8 criteria met—Stop; study design not feasible.
- 3-5/8 criteria met—Continue with modifications; feasible study design with modifications.
- 6-7/8 criteria met—Continue without modifications but monitor closely; feasible study design with close monitoring.
- 8/8 criteria met—Continue without modifications; feasible study design as is.

### Participant Timeline

We will instruct participants to participate in the discussion forum, at least, twice a week and in each weekly chat. In addition, we will instruct participants to access peer mentors selected per their own preference, at least, once every week. Participants can choose to access only one or several peer mentors, again at their own preference. Caregiver peer participants will be instructed to respond to messages directed at them from either mentors or other peers through the Web-based peer support site within 48 hours.

We will instruct peer mentors to participate in the discussion forums, at least, 2 times a week and in each weekly chat. In addition, we will instruct peer mentors to contact the research team if they are concerned about the well-being of participants. The research team will then contact those participants to assess the situation and recommend visiting their family doctor to access supports if required. We will instruct peer mentors to respond to messages directed at them through the Web-based peer support site within 48 hours.

### Sample Size

There has been no prior assessment of a peer support intervention for VAI caregivers on which to base our sample size calculations. Using feasibility criteria of 15% dropout and 70% participation in weekly chats, a sample size of 30 would allow us to be 90% confident that estimates are accurate within 22% and 28% percentage points, respectively [30].

### Recruitment

Recruitment procedures for the Web-based peer support program feasibility RCT are the same as those for the peer mentor training. We will aim to recruit 30 participants. We will recruit caregivers until we achieve the following minimum numbers: (1) ventilator subgroup (invasive: n=2; noninvasive: n=8); (2) diagnostic category (rapidly progressing disease: n=3; nonrapidly progressing disease: n=3; restrictive thoracic cage disorders: n=3); (3) relationship to care-recipient (spouse: n=4; child: n=4; parent: n=4); and (4) gender (male: n=5; female: n=7).

### Allocation

We will randomize consenting participants to the peer support intervention or waitlist control using a 1:1 ratio using Randomize.net. No stratification will be applied, and allocation will be concealed using opaque sealed envelopes. Those randomized to the waitlist control will be given access to the peer support program following the 12-week intervention phase. A waitlist control group was chosen as it is believed to be a cost-effective and ethical alternative to no-treatment control groups when primarily studying psychological and behavioral interventions such as the one in this study [31]. Those randomized to the intervention group will receive information through email about the website features (eg, forum, chat) and instructions in their use, log-in and profile instructions, and the weekly chat schedule. We will send weekly email reminders to encourage participants to access website resources, participate in the weekly chat, and draw their attention to active discussion forum threads.

### Data Collection

#### At Baseline

A blinded assessor will collect the demographic and health information from participants in both the intervention group and control group and administer the exploratory caregiver- reported measures (listed in Table 1) through email or over the phone (as per the participants’ preference). Our research team has previously used this battery of questionnaires identifying that completion takes approximately 30-40 minutes.

#### Upon Program Completion

We will ask participants (intervention arm only) to complete a program evaluation (through telephone or email, depending on preference). The program evaluation will assess the acceptability through a series of questions (eg, about the helpfulness of the program, how likable program was; 5-point Likert scale; generally, 1=very unacceptable; 5=very acceptable). The evaluation will assess satisfaction by asking participants the extent to which they agree with a series of questions about the program content, delivery, and outcomes (5-point Likert scale; 1=disagree; 5=agree) [45]. Finally, the evaluation will assess the usability by asking participants the extent to which they agree with a series of questions about the usability of various program features (5-point Likert scale; 1=strongly disagree; 5=strongly agree) [46]. We will invite participants to complete a semistructured qualitative interview to further explore their experience with the peer support program (eg, which features were most beneficial, what aspects were most challenging, what can be improved, what should be kept the same) and their perspectives on the support received from peers (eg, quality of support, influence on their caregiving experience). We will host 1-2 focus groups with the peer mentors using GoToMeeting. Using a semistructured interview guide, we will explore their experiences and perspectives of the Web-based program as trained peer mentors. Furthermore, we will audiorecord interviews and focus groups and transcribe verbatim.

### Statistical Methods

We will report descriptive statistics for demographic or health data, feasibility, and exploratory caregiver outcomes. To compare caregiver-reported outcomes between intervention and control groups, we will use the analysis of covariance with pretest scores as the covariate and group allocation, time, and the interaction between time and group allocation as independent variables. For nonnormally distributed scores, we will use a nonparametric alternative. All quantitative data will be analyzed in Statistical Analysis Software V9.4 (SAS Institute) using an intent-to-treat approach.

We will use the thematic content analysis to analyze interview and focus group transcripts following the framework outlined by Braun and Clark, which entails a line-by-line coding of transcripts, constant comparison, and generation of recurring themes [47]. We will use NVivo 11 software to facilitate the coding process. In collaboration with MBW, LR will analyze 20% of data to reduce bias and enhance the credibility and reliability of the qualitative findings [48].

**Table 1:** Exploratory caregiver-reported outcomes.

Measure name	Items	Score range	Description	Test-retest reliability (*r*)	Internal consistency (alpha)
Caregiving Impact Scale [32,33]	14	0-84	Higher scores suggest providing care interferes with caregivers’ abilities to maintain participation in valued activities	N/A^a^	.88 [32]
Barthel Index [34]	10	0-20	Higher scores indicate more functional independence	0.89 [35]	.87-.92 [36]
Pearlin Mastery Scale [37]	7	7-28	Higher scores indicate a greater sense of control over life	0.81 [37]	.75 [38]
Brief Coping Orientation to Problems Experienced (COPE) [39]	28	6-24 (problem-based coping); 22-88 (emotion-based coping)	Higher scores on either subscale represent greater use of that coping style	0.58-0.72 [40]	.57-.90 [39]
Personal Gain Scale [41]	4	4-16	Higher scores indicate caregivers’ discovery of inner strengths because of providing care	N/A	.9 [42]
Positive and Negative Affect Schedule [43]	10	10-50	Higher scores indicate more psychological well-being	0.47-0.68 [43]	.95 [42]
Centre for epidemiological studies short depression scale [24]	10	0-30	Higher scores sores indicate greater depression	0.41-0.70 [44]	.89 [42]

^a^N/A: not applicable.

## Results

We recruited 5 caregivers to be trained as mentors. The mentor training was completed in July 2018. We have recruited 4 caregivers to participate in the Web-based peer support program. We anticipate initiating the support program September 2018. Results are expected by early 2019.

**Figure 1:**
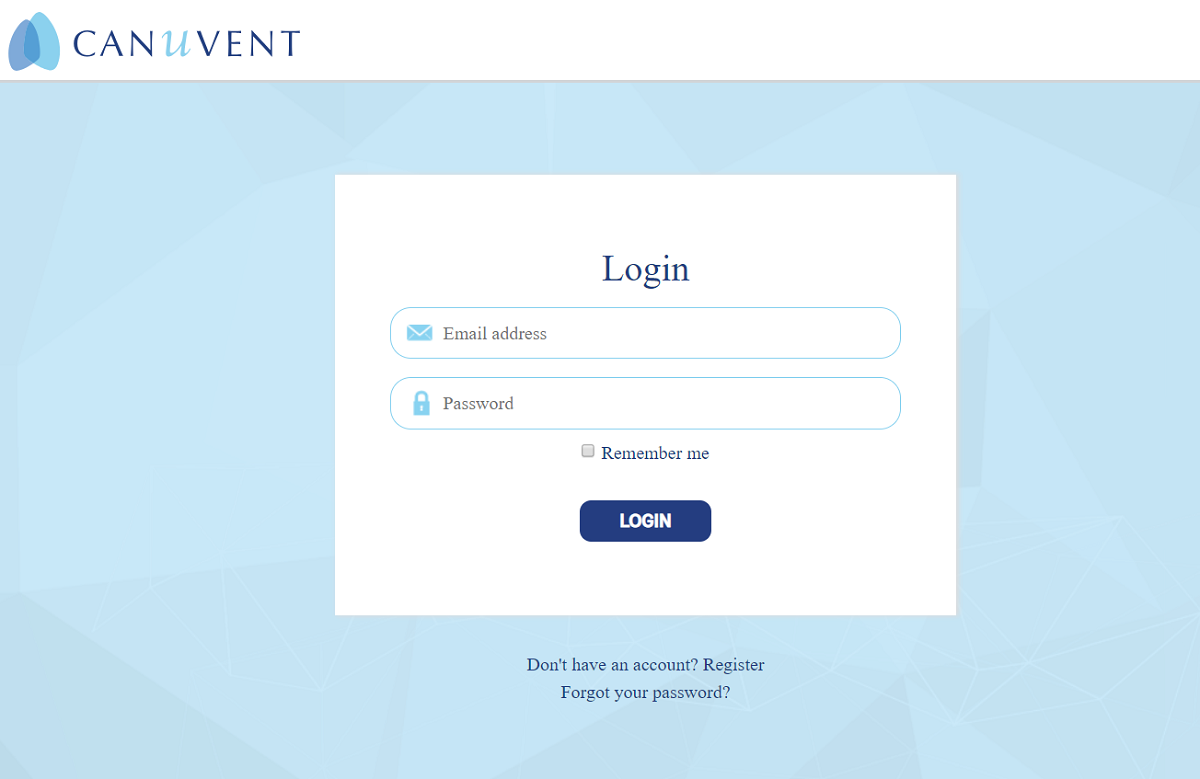
Screenshot of the initial log-in page.

**Figure 2:**
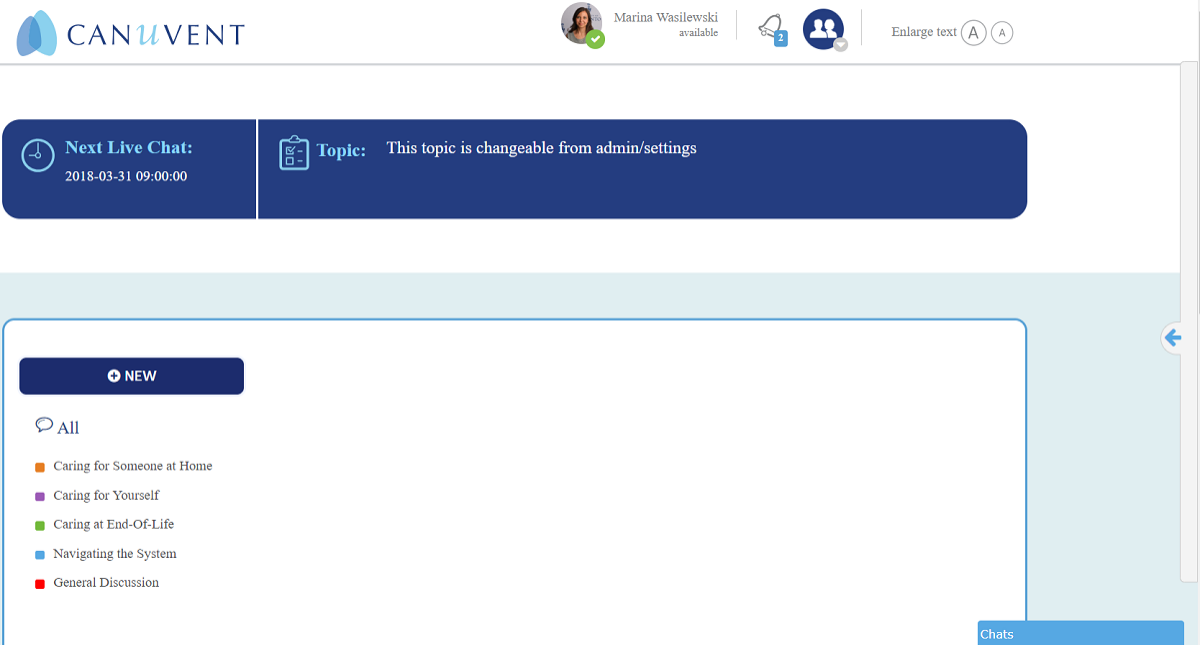
Screenshot of the home page.

**Figure 3:**
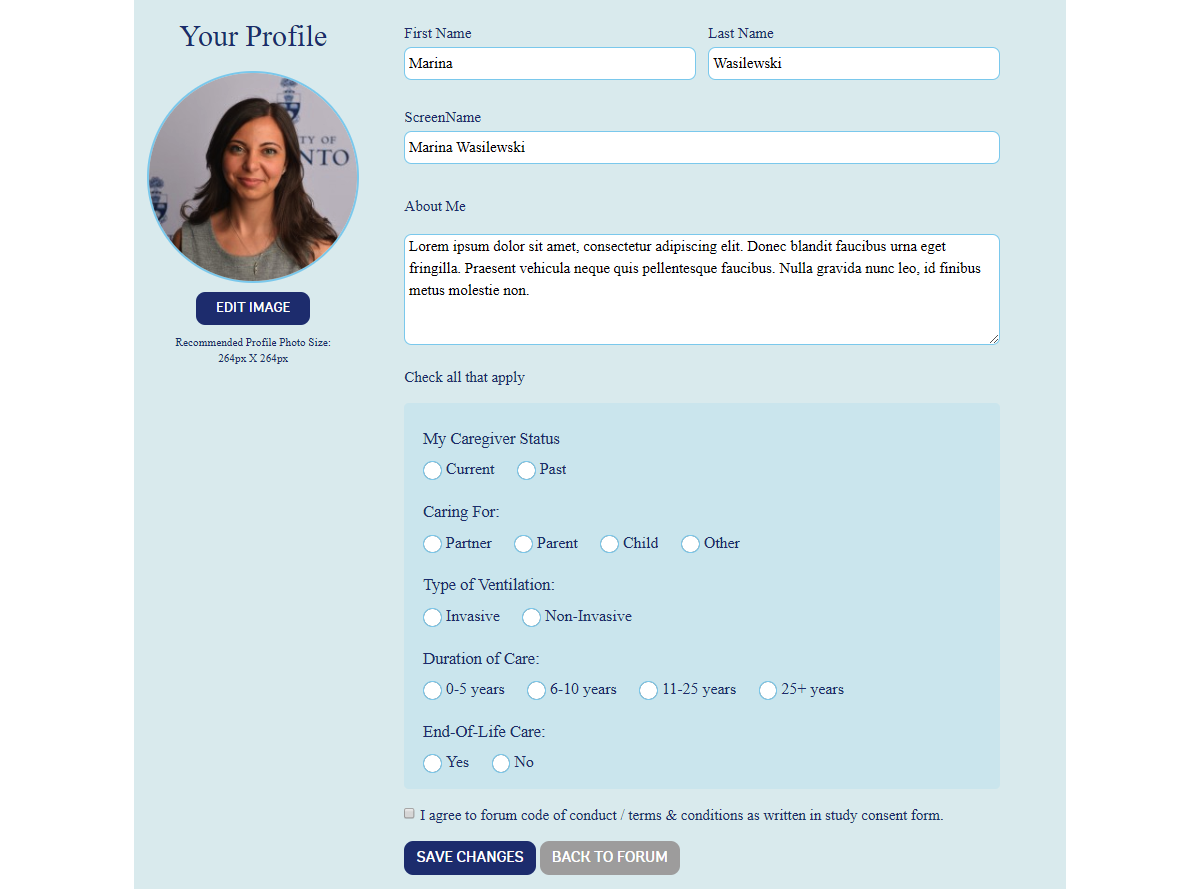
Screenshot of the profile set-up page.

**Figure 4:**
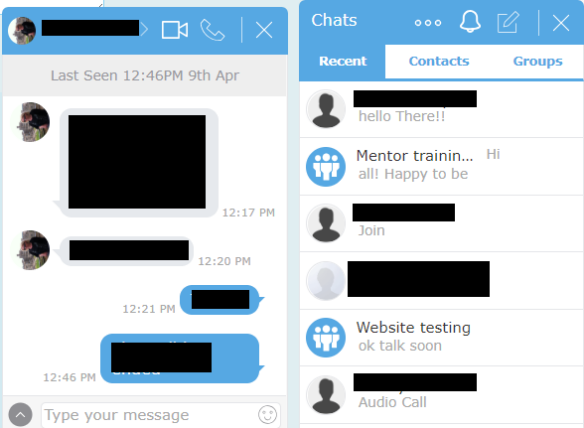
Screenshot of the private chat function.

Two caregivers have beta-tested the peer support website and issues identified have been addressed. Below are screenshots of the initial log-in page (Figure 1), home page (Figure 2), profile set-up page (Figure 3), and private chat function (Figure 4).

## Discussion

This study will result in the production and initial evaluation of a rigorously developed, evidence- and stakeholder-informed peer training and peer support program for caregivers of VAIs residing at home. Burdened and stressed caregivers may experience significant negative physical and emotional consequences to their own health, which may then impact their ability to care for VAIs who themselves are exceptionally vulnerable. Despite a growing body of evidence supporting the effectiveness of peer support interventions [20], there are currently no support programs of this nature tailored to caregivers of VAIs. The evaluation of the peer support program will highlight whether the inclusion of multiple communication tools is feasible, usable, and effective. The evidence generated will inform future iterations of the program so that it includes only the most valuable tools and optimizes them to enable support exchange.

The Web-based peer support program aims to empower community-residing VAIs and their caregivers to manage diseases necessitating mechanical ventilation. We anticipate that if caregivers have better health and quality of life, they will be better able to care for their loved ones who use ventilators. This can help VAIs remain in their homes and, thereby, mitigate the declines in health and quality of life associated with residential care placement [7].

Perceived strengths of this study include the intervention’s social networking-style interface that is likely to be familiar to participants, thereby enhancing the potential for greater usage and better usability of the peer support program. In addition, we anticipate that having multiple features on the website (eg, private chat, discussion forum, and live weekly chat) will increase the likelihood that participants will engage with and benefit from this peer support hub. Finally, the mixed-method nature of this research will not only provide insight into usage patterns and changes in health outcome scores but will also allow for an in-depth exploration of participants’ experiences with the websites and perceptions of how it has influenced their health and caregiving experience. As this is a feasibility study, it is not our objective to test significance. However, we are aware that this limits our ability to comment on the findings’ relevance and have identified it as a goal for future evaluations of the peer support program. While the inclusion of multiple Web-based features has the potential to increase engagement, there is still a possibility that the study will be limited by attrition.

## Abbreviations

HMV: home mechanical ventilation
RCT: randomized controlled trial
VAI: ventilator-assisted individual

## Appendix

Multimedia Appendix 1 Peer review report (for grant funding from Muscular Dystrophy Canada).
