# Supplementary material for: Development of a Web-Based Peer Support Program for Family Caregivers of Ventilator-Assisted Individuals Living in the Community: Protocol for a Pilot Randomized Controlled Trial
Source: JMIR Res Protoc. 2019 Feb 6;8(2):e11827. doi: 10.2196/11827 (PMC6386648; doi:10.2196/11827)
Supplement: Multimedia Appendix 1 [file resprot_v8i2e11827_app1.pdf]

October 19, 2016

Louise Rose, PhD
Sunnybrook Research Institute
2075 Bayview Ave. Room D108
Toronto, ON
M4N 3M5
Louise.rose@utoronto.ca

Re: Authorization of Funding – "**Development & Pilot Evaluation of an Online Peer Support Program for Family Caregivers of Ventilator-Assisted Individuals with Neuromuscular Disease Living in the Community**"

Dear Louise,

We are pleased to inform you that your grant application to our 2016 Respiratory Care Seed Grant competition was highly ranked and selected for funding. The budget allocation will be the full amount, as requested in your proposal. For your reference, attached is a summary of the feedback from the grant review panel.

Details of Grant
Period: 12 months
Start date: November 1, 2016*
End date: October 31, 2017
Amount: **$49,998**

To acknowledge acceptance of this grant, please complete the Authorization of Funding form and return it to my attention. *We require a signed copy of the Authorization of Funding by October 26, 2016 in order to release the funds to your academic/research institution.

If you have any questions, please don't hesitate to contact me. Congratulations to you and your team members!

Marla Spiegel
National Director, Research and Services

1-866-687-2538 ext 1103
Marla.spiegel@muscle.ca

Authorization of Funding

Lead investigator: Louise Rose, PhD
Project title: **Development & Pilot Evaluation of an Online Peer Support Program for Family Caregivers of Ventilator-Assisted Individuals with Neuromuscular Disease Living in the Community**
Grant Period: November 1, 2016 to October 31, 2017
Institution Paid: Sunnybrook Research Institute
Amount: $49,998

I, ____________________________ (Print Your Name), accept the following terms and conditions:

I commit to ensuring that the funds provided are utilized towards achieving the aims and outcomes as described in the application for funding.

I will advise Muscular Dystrophy Canada immediately regards to any funding overlap i.e. if I am in receipt or become eligible to receive any funding from another source for any part of this project.

I will adhere to Muscular Dystrophy Canada's payment and funding guidelines, and understand that Muscular Dystrophy Canada's will remit the funds for this project to my academic institution in trust. *(Please provide complete contact details for your institution's Finance/Grant Office below).*

I will acknowledge Muscular Dystrophy Canada's contribution in all written documentation and oral presentations related to this project, including scientific articles, news releases/conferences, public lectures and media interviews.

I will notify Muscular Dystrophy Canada prior to any news releases or public communications related to the results of this project.

I will provide a written report to Muscular Dystrophy Canada including a financial report, description of the outcomes of the project, and lay summary within 6 months of completion of the grant (deadline **March 31, 2018).**

______________________________________ ______________________________________
Signature of Lead Investigator Date

Mailing Address - Institutional Finance/Grant Office ____

____

Contact Name ________________________________ Title ________________________________

Phone (______) ________________________________ Email________________________________

The Lead Investigator must complete and return this form by October 26, 2016.
Attention: Marla Spiegel, National Director, Research. Fax: 416-488-7523. Marla.spiegel@muscle.ca

Peer Review Panel Summary Notes

Date: August 31, 2016

Title of Research Project	Development & Pilot Evaluation of an Online Peer Support Program for Family Caregivers of Ventilator-Assisted Individuals with Neuromuscular Disease Living in the Community
Name of Lead Investigator	Louise Rose
**Strengths of the proposal**	
The proposed work has broad application to many types of neuromuscular patient and is generalizable across Canada. Based on IT which is good for rural participation and is low cost for the user community. The proposal addresses both development *and* assessment of educational tools. The mentor training aspect is comprehensive, incorporating "emergencies". The study plan is solid, feasible and involves a strong collaborative approach. Ethics approval is already in hand. Implementation plans are developed in sufficient detail. The PI and team have a strong track record.	
**Weaknesses of the proposal**	
The focus is on caregivers, not patients. Some aspects of digital information security were unclear.<br>Wifi access could be a limiting factor for some families, thus not penetrating certain demographics.	
